# Natural Mordenite from Spain as Pozzolana

**DOI:** 10.3390/molecules25051220

**Published:** 2020-03-09

**Authors:** Leticia Presa, Jorge L. Costafreda, Domingo A. Martín, Isabel Díaz

**Affiliations:** 1 Instituto de Catálisis y Petroleoquímica, CSIC, c/Marie Curie 2, 28049 Madrid, Spain; leticia.presa.madrigal@alumnos.upm.es; 2 Escuela Técnica Superior de Ingenieros de Minas y Energía, Universidad Politécnica de Madrid, 28003 Madrid, Spain; jorgeluis.costafreda@upm.es (J.L.C.); domingoalfonso.martin@upm.es (D.A.M.)

**Keywords:** natural zeolite, mordenite, smectite, bentonite, pozzolanic cements

## Abstract

This work deals with anomalous concentrations of natural mordenite in the southeast of Spain. The X-ray diffraction (XRD) and scanning electron microscopy (SEM) studies evidenced that the samples contain mainly monomineral zeolitic phase of mordenite (70% to 74%), usually accompanied by smectite (montmorillonite), the principal component of bentonite. A study of the applicability of these zeolites is presented to establish the potential use as pozzolanic cements. For comparative purposes, synthetic commercial mordenite is also characterized and tested. The initial mixtures were prepared using cement and mordenite at a 75:25 ratio. Chemical analysis and a pozzolanicity test showed the high pozzolanic character. These mixtures were further added to sand and water, yielding the cement specimens to be used as concrete. Mechanical test results showed that the mechanical compression at 7 and 28 days fall into the range of 19.23 to 43.05 MegaPascals (MPa) for the cement specimens built with natural mordenites. The obtained results fall in the same range of cement specimens prepared with natural clinoptilolite, using mixtures within the European requirement for commercial concretes. Thus, these results and the low cost of natural mordenite of San José de los Escullos deposit supports the potential use of natural mordenite as pozzolanic cement.

## 1. Introduction

Cement is the major constituent material used in the production of concrete. The main component of Portland cements is clinker, a mixture of calcium silicates and aluminates obtained after sintering calcium carbonate at 1400–1500 °C, in a process that produces CO_2_ emissions to the atmosphere. The manufacturing of one ton of cement produces about one ton of CO_2_, with the construction industry being responsible for about 7% of all CO_2_ generated in the world. In addition, a large volume of raw materials is required to produce the billion tons of concrete required worldwide each year. The use of supplementary cementitious materials (SCMs) in the fabrication of concrete has proven to be a sustainable way to overcame economic, environmental, and technical disadvantages, while attaining reliable mechanical or durability properties. One way to reduce these emissions is to decrease the amount of clinker present in the cement by replacing part of it with pozzolanic materials with cementing characteristics such as natural zeolites. Zeolites can react easily with bases favoring the formation of silicates and aluminosilicates which are able to harden upon hydration. In cement chemistry, CSH and CAH represent hydrated calcium silicates and hydrated calcium aluminates that can be easily produced upon the reaction of zeolites with Ca(OH)_2_ yielding an amorphous aluminosilicate compounds called pozzolana.

Scientific knowledge and use of natural zeolites are fields in continuous expansion due to the appearance of new deposits or new applications. Mineralogical zeolitic varieties, such as heulandite and clinoptilolite, are commonly used in the manufacture of concretes, mortars [1], and in mixed mixtures of geopolymer mortars [2]. Natural zeolites produce profound effects on the hydration processes of cements [3] when they are used as supplementary materials to improve the quality of these binders [4]. Natural zeolites are known to produce a plasticizing effect to the cementitious mixture [5] and notably reduce the alkali–silica reaction as well as the damaging effects of sulphates [6]. In comparison with other pozzolanic materials, such as metakaolin, zeolites provide better properties to the durability of mortars and concretes, while their treatment is more economical and hardly produces environmental impacts [7].

In this work, the intention is to contribute to bust mordenite (MOR according to the three-letter coding of the International Zeolite Association) as a relevant component in these uses, together with heulandite and clinoptilolite. In particular, the mordenite we dealt with is found in a deposit in the southeast of Spain.

Spain does not have large deposits of natural zeolites, despite its great geological variety and its geographical extension. Only a few previous studies published, mostly in Spanish, mentioned the presence of zeolites in outcrops of volcanic rocks of the Iberian Peninsula and in the Canary Islands. The study area is in Cabo de Gata, Almería, Spain, at 2°4′08″ west longitude and at 36°46′42″ north latitude. The dimensions of the deposit fluctuate between 350 × 200 m. There are two areas of exploitation: The first is in the north, while the second is in a south–southwest direction. The thickness of the mineralized zone is estimated at more than 50 m. The exploitation of the quarry began around 40 years ago and is commonly commercialized as bentonite.

The application of natural zeolite in the manufacturing of pozzolanic cements has been known since the Roman Empire, however, the first scientific reports are from the early 20th century, and show a growing trend in the late 90s until recent decades [8]. Clinoptilolite is the most commonly used commercially available natural zeolite, given its good ion exchange properties. A large number of publications studied the pozzolanic activity of clinoptilolite compared to other substitutive additives due to its reactivity [9,10], allowing the introduction of very high ratios of zeolite in the cement mixtures while maintaining good properties [11,12]. The potential of natural clinoptilolite as an additive on cement is still investigated nowadays, showing that the addition of 5–10% clinoptilolite as the cement substitute improves the floatability, cohesion, adhesiveness of mortar and the 90-day compressive strength of mortar [13].

However, the occurrence of mordenite deposits [14] awoke interest of this versatile natural zeolite as a component of environmentally friendly cements [8,15,16]. The emission of CO_2_, produced by the manufacturing of cement (estimated 756 kg of CO_2_ per ton of clinker), was decreased by replacing part of the cement with zeolites, so that by using a mixture of natural zeolite and cement, “green concrete” is obtained, for which the manufacturing processes show a lower impact on global warming than those of ordinary Portland cement [17]. 

In this direction, our aim is to obtain concrete based on natural mordenite (MOR) from Spain, using ratios according to the European regulations UNE-EN 196-1:2011. By replacing 25% of the 22.8 Mt of cement feasible for commercialization in Spain in 2020, [18] the CO_2_ emissions could be reduced by 23% (approximately 4 Mt/year). 

## 2. Results

### 2.1. Characterization of Natural and Synthetic Mordenites

**Figure 1:**
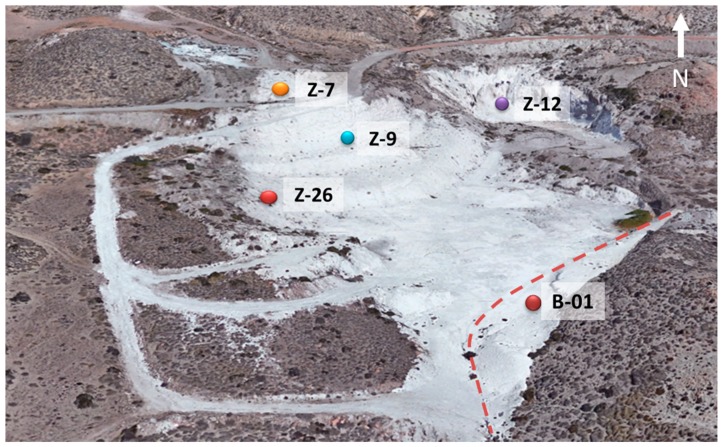
Photograph of the San José-Los Escullos deposit (google earth) where the natural mordenite samples are collected, the actual location of each collected sample is indicated.

The natural mordenite samples to be studied were taken from different points of the San José-Los Escullos deposit. The four natural samples were collected through manual sampling of lithic fragments of outcrops. Samples labeled Z-9 and Z-12 were collected in the north area of the San José-Los Escullos deposit, where the presence of zeolitic rocks was clearly identified given the sharp interaction with the host rocks. On the other hand, samples Z-7 and Z-26 were obtained in the central part of the deposit as indicated in Figure 1.

**Figure 2:**
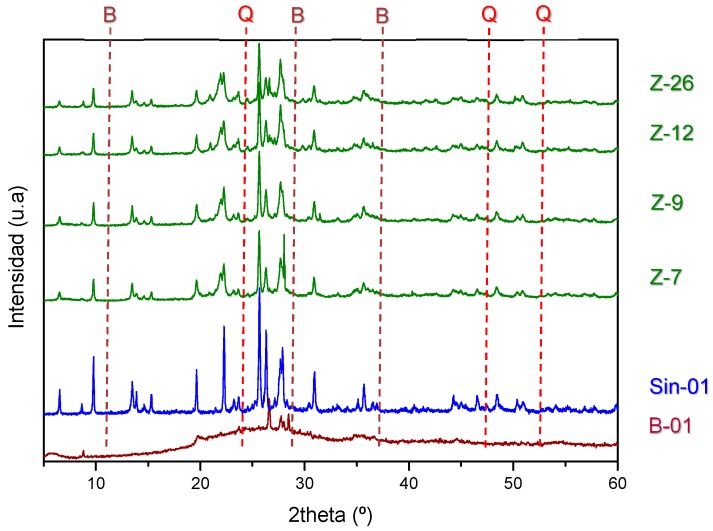
X-ray diffraction patterns of the natural mordenites from Spain (Z-7 to Z-26), commercial synthetic mordenite (Sin-01), and the bentonite host rock (B-01). Other mineral phases of bentonite (smectite) are market with “B” and quartz with “Q”.

The identification of the phases is carried out by X-ray diffraction (Figure 2), and the resulting profiles are compared to that of a commercially available synthetic mordenite (CBV10A), labeled Sin-01. In addition, a sample of natural bentonite, labeled B-01, was taken from the host rock for comparison purposes.

The analysis of the X-ray diffraction showed very similar profiles among the natural mordenite samples, composed mainly of MOR structure (according to the three-letter coding of the International Zeolite Association), with impurities of smectite (marked with “B” in Figure 2) and quartz (“Q” in Figure 2). These impurities are not shown in the synthetic mordenite because of its monomineral composition. The four samples follow a similar trend, showing no apparent differences in composition nor crystallinity or purity, however, further analyses of the intensities will allow estimation of the percentages of phase purity (Table 1). On the other hand, sample B-01 reveals a typical profile of smectites of the montmorillonite type. This profile is characteristic of smectite, given the amorphous broad baseline, which is also present in the natural mordenite samples, not in the synthetic one. This coexistence in the natural samples indicates a possible complex paragenesis, meaning that both mineralogical phases were generated at the same geological time following the same geological formation process; there is a unique geological origin of both species. This process was not observed for the natural mordenites identified in deposits of Ethiopia [19].

The relative purity in the natural samples has been calculated using the areas of the diffractogram of the different mineralogical phases. The MOR phase was clearly identified, allowing the phase quantification using the addition of all the peaks areas belonging to MOR phase. On the other hand, for smectite and quartz, only three peaks were identified for each phase (marked in Figure 2 as “B” and “Q”, respectively) while there was a wide number of small peaks and noise remaining unidentified hindering a more precise phase purity calculation.

The unit cell parameters obtained from these X-ray diffraction (XRD) calculations are included in Table 1. As it can be observed, the unit cell dimensions coincide with the characteristic parameters of mordenite obtained from the Handbook of Natural Zeolites (a = 18.052–18.168 Å; b = 20.404–20.527 Å; c = 7.501–7.537 Å) [14]. The unit cell calculated for the synthetic mordenite studies in the work is also included.

The surface area of the richest MOR sample was measured. The obtained result for sample Z-7 is rather high compared to other natural zeolites [20] yet much smaller than the textural properties of synthetic zeolites.

As it can be observed, the sample purity decreases from Z-7 to Z-26, with Z-7, Z-9 and Z-12 above 70%, which is a considerably high value for a natural zeolite deposit. This tendency could be explained based on the location within the deposit because the concentration of mordenite in the deposit decreases as the hydrothermal solutions approach the host rocks, which consist of pyroxenic andesites, dacites, and dacite tuffs. Thus, there is a marked zoning in the mineralization, characterized by an anomalous concentration of monomineral mordenite in the central part that gradually decreases towards the flanks. With the decrease of the mordenite content, there is an increase of the smectites at the same time [21]. According to Figure 1, samples Z-7 and Z-9 have been collected from the central part of the deposit, while Z-12 and Z-26 are closer to the host rock, with Z-26 being the least pure sample, and the least crystalline being Z-12. These results are consistent with further systematic studies regarding the purity of the deposit previously conducted by Costafreda et al., yet they are reported in Spanish [22]. These studies confirmed the orientation of the deposit, in which the process of zeolitic mineralization is due to the irruption of hydrothermal solutions in the central part of the deposit, where several systems of faults and diaclases converge.

**Figure 3:**
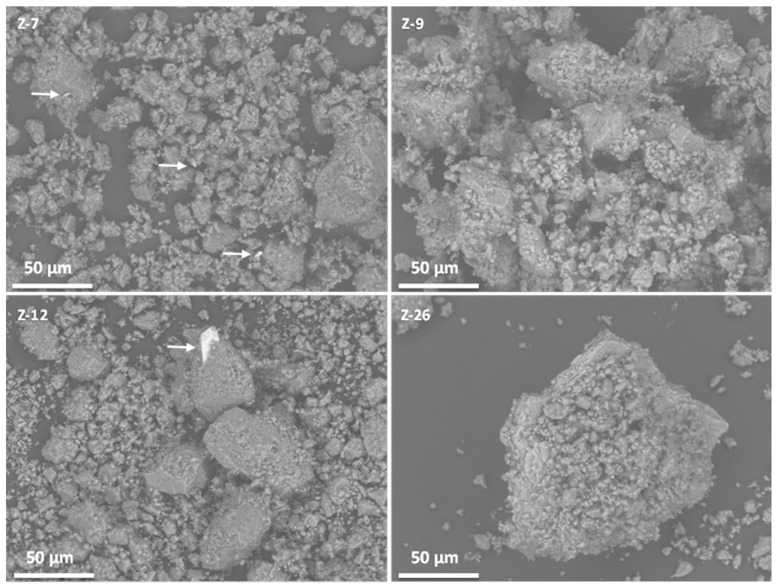
Scanning electron microscopy (SEM) micrographs of natural mordenites. White arrows indicate Fe-based impurities.

The morphological studies of the samples were conducted by scanning electron microscopy in order to survey the homogeneity of the identified natural mordenites from Spain. Figure 3 collects representative micrographs of the four natural mordenites.

**Figure 4:**
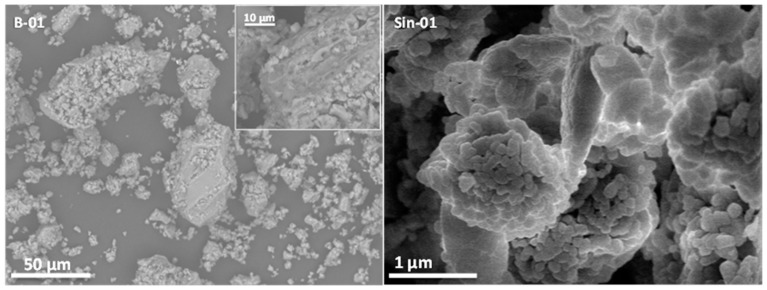
SEM micrographs of bentonite B-01 sample and synthetic mordenite (Sin-01).

Sample Z-7 shows a relatively homogeneous dispersion of crystals in the range 10 to 20 µm, with the random presence of larger agglomerates with elongated dimensions of 50 to 100 µm. The presence of high atomic number phases could be observed as bright spots in Z-7, since the scanning electron microscopy (SEM) microscope employed used a backscattered detector. These bright spots, marked with white arrows in Figure 3, are related to high content Fe phases. Z-9 shows similar 10–20 µm agglomerates, although the larger agglomerated particles are more abundant. Energy dispersive X-ray spectroscopy (EDX) studies do not univocally allow the distinguishing of the mordenite phase since the composition of smectites is very similar, and moreover, the morphology of the B-01 sample did not reveal any particular particle shape in the microscope employed for this work (Figure 4). Z-12 shows similar results with two ranges of particle sizes, with a higher presence of the larger ones. In this particular case, the brighter crystals related to the Fe-content phases appear larger. The EDX of these brighter crystals reveal Fe and Si ratios that could be tentatively assigned to the ilmenite phase [23], anyhow, this phase is only observed in traces. Finally, Z-26 clearly shows large agglomerated crystals as it is expected from the lower purity percentage.

Further studies in the B-01 sample (Figure 4) allowed tentative assignment of these larger agglomerates as the bentonite phase. Although the typical lamellar particle morphology of bentonite could not be clearly identified in this sample, probably due to the low resolution of the microscope, a high magnification revealed a layered structure of the edges of these large particles. Furthermore, a systematic EDX analysis of these larger agglomerates showed a tendency towards higher K content, which is more abundant in bentonite than in mordenite.

Finally, synthetic mordenite (Figure 4) is composed of crystals as small as 0.1 µm homogeneously shaped forming orthorhombic particles.

Table 2 shows the results of the chemical analyses of the natural mordenites. These results allow the estimation of the composition; however, the overlapping of chemical composition among the phases present in the samples hinders the further calculation of MOR percentages. Nevertheless, the obtained results fall in the range expected for mordenite-rich samples with a Si/Al ratio of 5.

**Figure 5:**
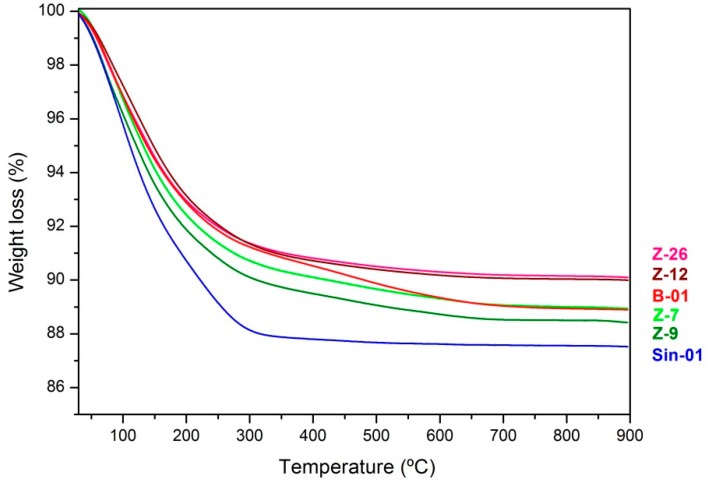
TGA curves of natural mordenites, synthetic one (Sin-01) and bentonite (B-01).

Thermogravimetric studies were also carried out due to the relevance of the water content to the application in cements. Total weight loss has been included in Table 2, and the TGA curves are plotted in Figure 5.

The profiles in Figure 5 show a main first thermal process occurring between 25 and 350 °C corresponding to a rapid incipient loss of surface moisture of the sample, followed by the dehydration of the cavities of the zeolite, with a mass loss almost greater than 7% in all cases. The behavior of the curve shows a process in which the loss of mass is continuous and almost instantaneous, in which all the physiosorbed phases are desorbed from the zeolitic porous structure.

Above 350 °C, the differences between the samples are more evident, indicating the loss of water from the constitution of smectite. Condensation of silanol groups contributed to this event with a very slow mass loss due to the loss of water from the constitution and the decomposition of the sample. The loss of mass is less pronounced and can reach 1%, the structural stability limit of synthetic mordenite. Between 700 °C and 900 °C, there is a normalization of the curve, in which no loss of mass is determined, which corresponds to the structural rearrangement in post dehydration/dehydroxylation conditions with no compositional changes.

The values represented in Table 2, and extracted from Figure 5, indicate that Sin-01 synthetic zeolite has the highest value of loss of ignition, i.e., the highest value of water content released upon heating at 900 °C. This can be explained by a greater pore volume due to the purity of this sample, being formed only by a microporous solid with a greater capacity to accommodate water molecules and cations in general. Synthetic mordenite shows no thermal event above 350 °C due to the homogeneity of the samples. Undoubtedly, synthetic zeolite has a very uniform crystalline structure that allows greater adsorption–absorption of molecules, which at the same time means a greater capacity for desorption. On the contrary, if we compare this curve with that shown for the bentonite sample (B-01), it is evident that there is a significant contribution in this case in the range of 350–550 °C which corresponds to the water of constitution of smectites. This event indicates the different nature of this sample as compared to zeolites. The presence of a small contribution of this thermal event in the curves of the natural zeolites corroborates the presence of impurities in the four samples. This trend is observed in the rest of the natural zeolite where the similarity in the curves indicates a paragenesis between the smectites and the natural mordenites of Cabo de Gata. The total amount of water loss in the natural zeolite samples is directly related to the purity of the sample, with the Z-7 samples (74% purity) showing the largest mass loss after Sin-01, and the Z-26 samples (67%) showing the lowest, as shown in Table 2.

### 2.2. Characterization of the Pozzolanic Cement

In order to evaluate the properties of these zeolites as pozzolana, mixtures of 75/25 cement/mordenite were prepared according to the European Standard [24]. Table 3 shows the results of the chemical analyses carried out on the cement sample mixture following the abovementioned standards regulation, described in the materials and methods section.

These results are indicative of the final reactivity of the cement, and therefore, the composition varies as a function of the active additives added to the mixture. Since the percentage of additive in the mixture is constant (25%), the variations will be directly related to the nature of the zeolite sample employed. The samples are rich in silica and alumina, as expected, given the zeolite composition. When the mixture contains 25% of synthetic mordenite (Sin-01), the reactive SiO_2_ reaches the highest value, 73%, which is 98.6% of the total SiO_2_ of the mixture. This means that virtually all the silica in the sample reacts with the cement while keeping the MgO, CaO, and Fe_2_O_3_ in low values, as indicated by the European Standard Regulations [25]. Natural samples contain 1–3% less total SiO_2_ but the decrease in the reactive SiO_2_ shows 10% to 15% decrease due to the impurities present in these samples that hinder the reactivity. Surprisingly, the sample with the highest content of reactive SiO_2_ is Z-26, with 63%, which represents 88% of the total silica, while these samples showed the least purity (67% in mordenite). On the other hand, Z-7, being the purest sample, shows the lowest reactive SiO_2_ (59%, 82% of the total SiO_2_). This explains the high percentage of insoluble residue observed. The XRD of the sample Z-7 allowed for identifying strong peaks of quartz, for which the crystalline structure is very strong, hindering the reactivity and therefore appearing as an insoluble residue. However, as we will see in the results of compressive strength, this factor does not negatively influence the final properties of the cements because it is a resistant and stable material which does not produce further reactions with other components of the cement leading to changes in its composition or properties.

Free CaO and free MgO react with water to form Ca(OH)_2_ and Mg(OH)_2_ which take up more space than the original oxides, therefore they can cause harmful expansions; consequently, all cement standards limit the contents of free CaO and MgO. The results obtained by the natural zeolite samples were below these limits, so it can be concluded that the cement made with these samples will not be damaged by the expansions produced by the presence of these oxides.

**Figure 6:**
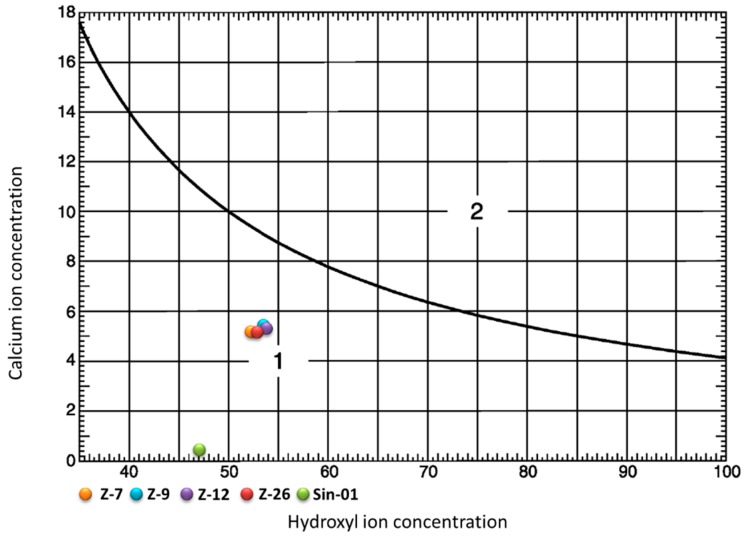
Curve for the results of pozzolanic test.

The pozzolanic activity was determined by comparison between the amount of Ca(OH)_2_ contained in an aqueous solution, in contact with the hydrated cement, and the amount of Ca(OH)_2_ necessary to obtain an aqueous saturated solution with the same alkalinity as the previous one. The test is considered positive if the samples are positioned below the isothermal of solubility at 40 °C in the graph (plotted in Figure 6), which means that the concentration of Ca(OH)_2_ in solution is lower than the saturation concentration. The studied samples were tested according to the European Standard Regulation [26].

In the pozzolanic activity test, all samples are below the isotherm of solubility at 40 °C, which involves a high pozzolanic character of the samples tested. The synthetic mordenite (Sin-01) has a behavior towards magnitude 0 of calcium oxide concentration (mmol/L), which indicates the ability of the zeolite to react quickly with the cement in solution. The natural zeolite samples have a strong similarity in pozzolanicity given their position in the graph, maintaining all of them a positive value.

### 2.3. Appplication as Concrete

**Figure 7:**
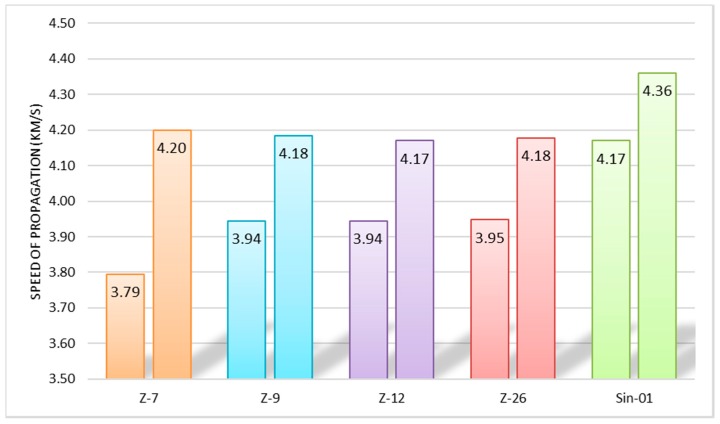
Results of ultrasonic pulse velocity (km/h) of the specimens at 7 and 18 days of age.

The Ultrasonic Pulse Velocity (UPV) method was used for ultrasound analysis, which provides the time it takes for a wave to travel a distance through the specimen. Knowing the time readings and the travel distance of the waves, the speed of the ultrasonic waves was calculated. Figure 7 shows the results of the ultrasound analyses of specimens at the age of 7 and 28 days.

In all samples, an increase in the speed of the ultrasonic waves is observed as the setting time increases. This behavior may be related to the removal of water that occurs as the setting of the specimen progresses, which favors the increase of the mineral phase [27]. The purest natural zeolite, the Z-7 sample, shows the highest increase in the speed of ultrasonic waves which is related to its pozzolanic characteristics that increase its mechanical resistance as the setting progresses. The synthetic sample shows higher ultrasonic wave velocity values than those obtained from natural samples. This tendency denotes a higher consistency of the specimens, which is related to better mechanical properties and less possible structural defects in the specimen.

**Figure 8:**
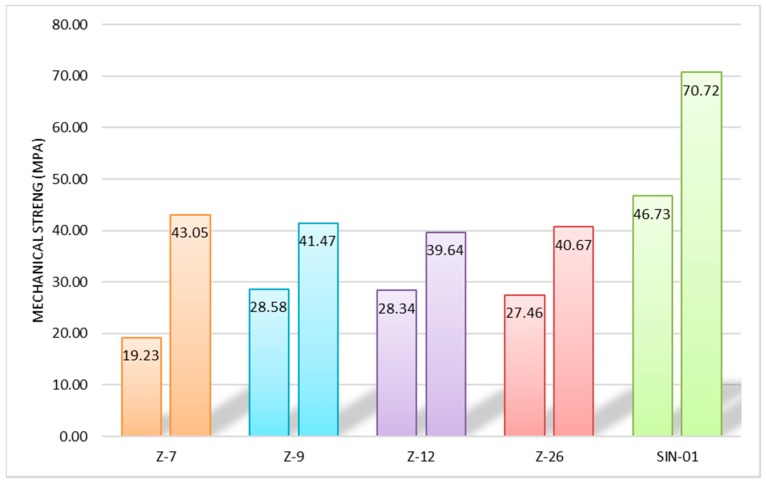
Results of mechanical resistance of the specimens at age of 7 and 28 days.

The compressive strength was performed on the specimen half-prisms resulting from the bending strength to indicate the maximum stress a material can support under a crushing load until it breaks. This is calculated by dividing the maximum load by the original cross-sectional area of a specimen in a compression test, following the indications of the European Standard [18]. Figure 8 shows the results of the mechanical strength resistance (MPa) analyses of specimens at the age of 7 and 28 days.

Initial and final setting times of the pastes were determined with the Vicat needle test according to the European Standard [28]. For the determination of the setting times, the pastes were mixed on 75/25 cement/zeolite ratio. To evaluate the setting times (Table 4), two repetitions were performed on the natural mordenite samples and another two on the synthetic sample, to study the reproducibility of the setting times. The results obtained in the synthetic mordenite are significantly lower than those obtained in the synthetic mordenite. Moreover, the repetition of the setting time indicates the stability of the properties of the synthetic mordenite associated with its homogeneity.

## 3. Discussion

The results presented above, obtained for the characterization of the natural mordenite samples through X-ray diffraction and scanning electron microscopy, indicate a mineralogical composition consisting of the main phase of mordenite and a secondary phase consisting of smectite and quartz and small quantities of metallic minerals. Despite knowing the percentage of the phases identified as impurities present in the samples, it is known that the natural samples have a complex composition, mainly formed by mafic and felsic minerals, such as amphibol, pyroxene, biotite and feldspar, followed by minor amounts of quartz, opal-CT/cristobalite, and smectite among others [29], which makes their quantification difficult. The sample with the highest natural mordenite content is Z-7, collected from the central part of the deposit. Thermogravimetric analyses show a typical behavior of the mordenite. The determination of pozzolanicity indicates that the studied samples are materials with high pozzolanic reactivity fundamentally concerning synthetic mordenite. The results obtained by the natural and synthetic samples in the applicability test indicated that the performance offered by the synthetic mordenite exceeds that of the natural samples, probably due to the smaller crystal size and the absence of impurities in this sample. However, it is expected that at higher setting ages the values of natural and synthetic mordenite will get closer and equal. Nevertheless, the difference does not justify the necessity for a commercial synthetic zeolite in this particular use, where a low cost raw material, a natural zeolite, could be used yielding similar properties.

## 4. Materials and Methods

### 4.1. Description and Characterization of Mordenite Samples

The tested samples were composed of four natural samples taken from different points of the San José-Los Escullos deposit and one synthetic mordenite sample. The four natural samples were taken through manual sampling of lithic fragments of outcrops. Samples labeled Z-9 and Z-12 were collected in the north area of the San José-Los Escullos deposit, where the presence of zeolitic rocks was clearly identified given the sharp interaction with the host rocks. On the other hand, samples Z-7 and Z-26 were obtained in the central part of the deposit, as indicated in Figure 1. The natural mordenite samples were granulated before further analyses (Figure S1). In a first step, a jaw crusher was used to obtain grain size below 3 cm. Then, a second jaw crusher reduced to a maximum 1 cm, and finally, a vibrating-disc mill was employed to obtain a Blaine specific surface of 4000 ± 200 cm^2^/g, which was the same specific surface that the cement reference.

Synthetic mordenite was purchased from Zeolyst, code CBV10A, supplied in fine powder form which does not require further granulation particle treatment.

In addition, a sample of natural bentonite labeled B01, was taken from the host rock for comparison purposes.

### 4.2. Characterization Techniques

X-ray diffraction was performed in a Philips diffractometer X′ PERT using the radiation CuKα (λ = 0.154 nm). The percentage of mordenite present in the samples was calculated by measuring the area of the main mordenite peaks concerning the total area of the diffractogram of each sample. Nitrogen adsorption–desorption isotherms were measured at −196 °C using the micrometrics ASAP 2420 and BET calculation. Scanning electron microscopy studies were performed on a Hitachi TM-1000 Tabletop equipped with EDX. In these cases, the samples were observed without coating. A higher resolution SEM was employed to image synthetic mordenite. A Philips XL30 S-FEG was employed using Cr coating of the sample (Quorum. model Q150T s). X-ray fluorescence was carried out using a Philips WDXRF spectrometer (PW 1404) equipped with a collimator to reduce the angle of divergence of the x-rays. A Perkin-Elmer TGA7 thermobalance was used for the thermogravimetric analyses in a range of 25 to 900 °C using a heating ramp of 20 °C/min in an atmosphere of air.

### 4.3. Characterization of the Pozzolanic Cements

The mixture used in the characterization of the pozzolanic cement was composed of 75% of a reference cement type II 42.5R and 25% of mordenite. The chemical analyses of the mixtures were carried out following the European Standard Regulations [24], which specifies the procedures and the materials to be used. The pozzolanicity tests were conducted according to the European Standard [25], which specifies the procedures and the materials to be used.

### 4.4. Appplication as Concrete

Tests carried out to determine the application of the study material as concrete was conducted on prismatic specimens of 40 × 40 × 160 mm composed of 400 g of mixture, with a 75/25 ratio of cement/zeolite, 225 g of standardized CEN sand, and 225 of filter water (water/cement ratio 0.50). The cement used was a reference cement type II 42.5R.

The preparation of the cement specimens was carried out by the European Standard [18].

The determination of the speed of ultrasound propagation was carried out following the European Standard [27] according to the equation:

Speed of propagation (m/s) = distance (km)/time (s)

A CONTROLS-UPV E48 ultrasound equipment was employed using Ultraschall-Gel Sauerland as a contact gel.

The bending resistance test was carried out following the European Standard [18]. The three-point load method has been used with a MEM-101-FX bending press. To carry out the compression test, the indications of the European Standard [18] were followed. The resulting half-prisms from the bending test were tested in an ELE/SDE compression press, with an Ibertest compression device, with a uniform load increase of (2400 ± 200) N/s during the whole application time until breakage.

The initial and final setting processes were tested under the specifications set out in the European Standard [28] with the manual Vicat instrument.

## 5. Conclusions

The results of the chemical analysis of the mixture made of 75/25 cement/zeolite ratio of the mordenite samples both natural and synthetic allow us to conclude that they are suitable for use as additives in the production of pozzolanic concrete because their values are within the limits established by the regulations. However, an exception is the value of the insoluble residue determined in the samples of natural mordenite due to the presence of non-reactive silica. This does not adversely affect the durability of the concrete. The determination of pozzolanicity indicates that the Spanish natural mordenites are materials with high pozzolanic reactivity. The tests carried out on the mixture of cement and mordenite have allowed us to determine the behavior of the samples in their final application, showing that despite containing impurities and the difficulties of characterizing natural samples, these are perfectly valid as active additives in cement.

## Figures and Tables

**Table 1:** Percentage of mineralogical phases obtained for the natural zeolite samples as a result of the estimations calculated from the X-ray diffraction (XRD) profiles. Unit cell parameters calculated for the natural mordenite phase is also included.

Sample	MOR (%)	Impurities (%)	*a* × *b* × *c*	Surface Area (m^2^/g)
Z-7	74	26	18.09 × 20.48 × 7.51	77
Z-9	71	29	18.10 × 20.47 × 7.51	
Z-12	71	29	18.07 × 20.40 × 7.51	
Z-26	67	33	18.08 × 20.46 × 7.51	
Sin-01	100		18.02 × 20.40 × 7.51	425

**Table 2:** Chemical composition of the natural mordenite samples obtained from X-ray fluorescence.

Sample	% Oxides Weight								Wt los ^1^%	Si/Al	Si/ (Al + Fe)
	SiO_2_	Al_2_O_3_	CaO	Na_2_O	K_2_O	MgO	Fe_2_O_3_	TiO_2_			
Z-7	68.30	11.95	1.15	2.89	1.38	1.27	1.56	0.08	11.06	5.0	4.3
Z-9	68.42	9.64	1.2	3.63	2.14	1.09	1.27	0.11	11.58	5.13	4.5
Z-12	67.04	10.09	2.3	2.05	1.98	1.56	1.56	0.11	10.01	5.3	4.3
Z-26	63.84	13.07	1.99	2.85	2.39	1.43	1.64	0.11	9.91	5.13	4.3
Sin-01									12.48		
B-01									11.11		

^1^ Weight loss at 900 °C calculated from the thermogravimetry TGA profiles.

**Table 3:** Results of chemical analysis of the mixtures of mordenite and cement.

Sample	Z-7	Z-9	Z-12	Z-26	Sin-01
Total SiO₂	71.3	72.02	72.28	71.8	74.01
MgO	0.77	0.87	0.8	0.88	0.13
CaO	0.88	1.45	1.24	1.35	0.81
Fe₂O₃	1.15	1.21	1.24	1.39	0.48
Al₂O₃	11.82	11.67	11.58	11.84	9.69
^a^ Reactive SiO₂	58.68	61.57	62.12	63.16	72.98
^b^ IR	19.87	16.49	16.83	14.08	1.82
^c^ LoI	5.96	5.97	6.02	6.35	7.85

^a^ Reactive SiO_2_ is the part of total silica that reacts with calcium oxide (CaO) to produce belite (2CaO-SiO_2_) and alite (3CaO-SiO_2_), two of the four major minerals in cement. It is determined by colorimetry [24]. ^b^ IR is the insoluble residue obtained after treatment with HCl and boiling KOH. It is determined by weight [24]. ^c^ Loss of ignition at 950 ± 25 °C.

**Table 4:** Results of setting time of the specimens.

Specimens	Z-7	Z-9	Sin-01A	Sin-01B
Initial setting	165	195	105	105
Final setting	235	260	180	180

## Supplementary Materials

Figure S1: Images of (a) grinding to < 3 cm (b) grinding to < 1 cm and (c) milling machines.
