# Supplementary figures and images for: Natural Mordenite from Spain as Pozzolana
Source: Molecules. 2020 Mar 9;25(5):1220. doi: 10.3390/molecules25051220 (PMC7179431; doi:10.3390/molecules25051220)

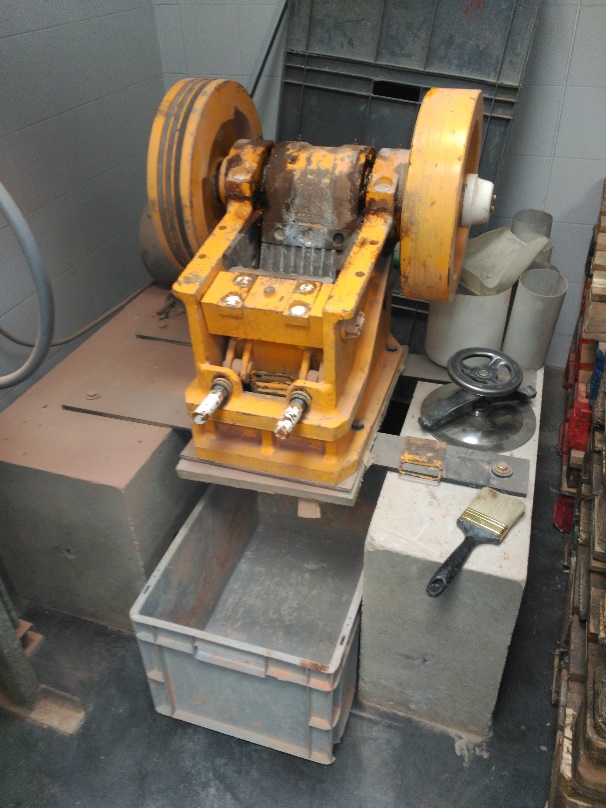

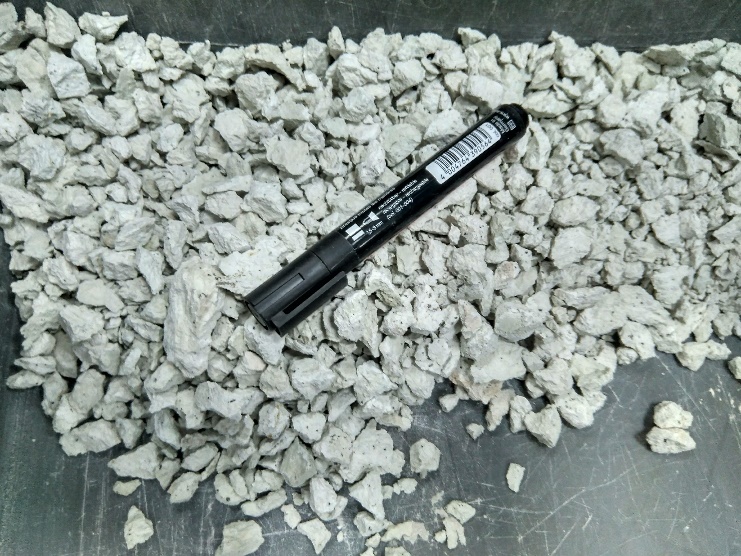


**(a)**


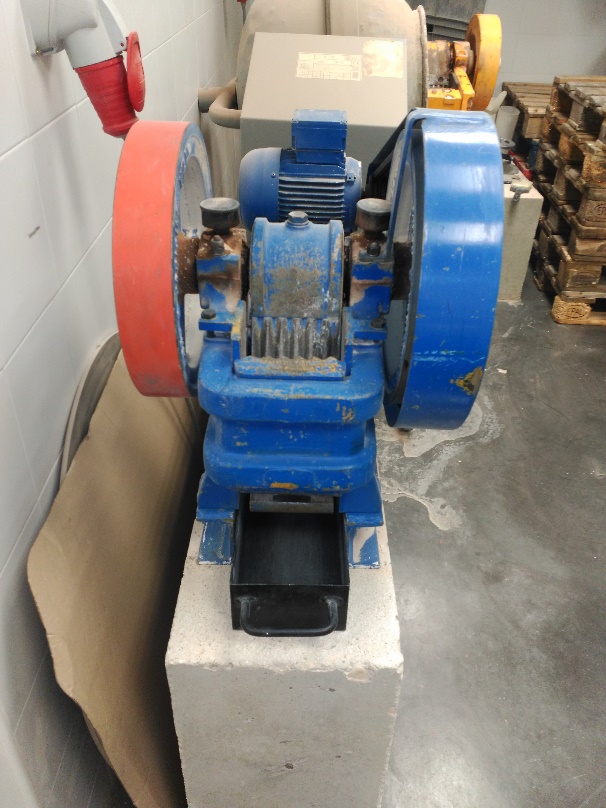

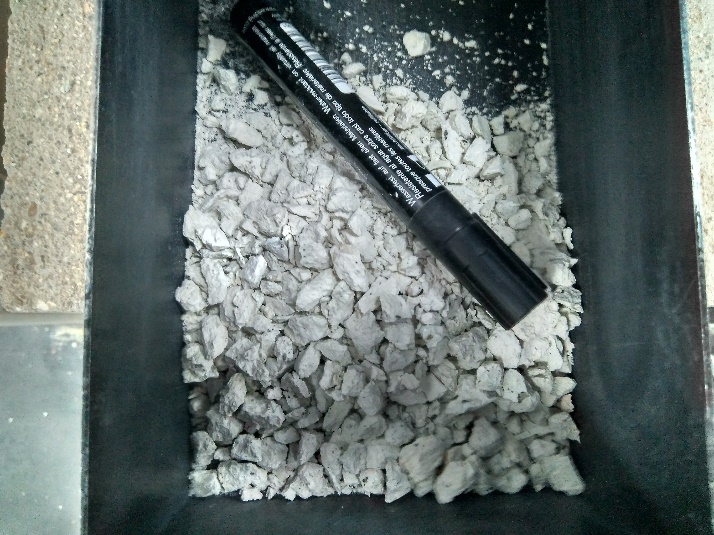


**(b)**


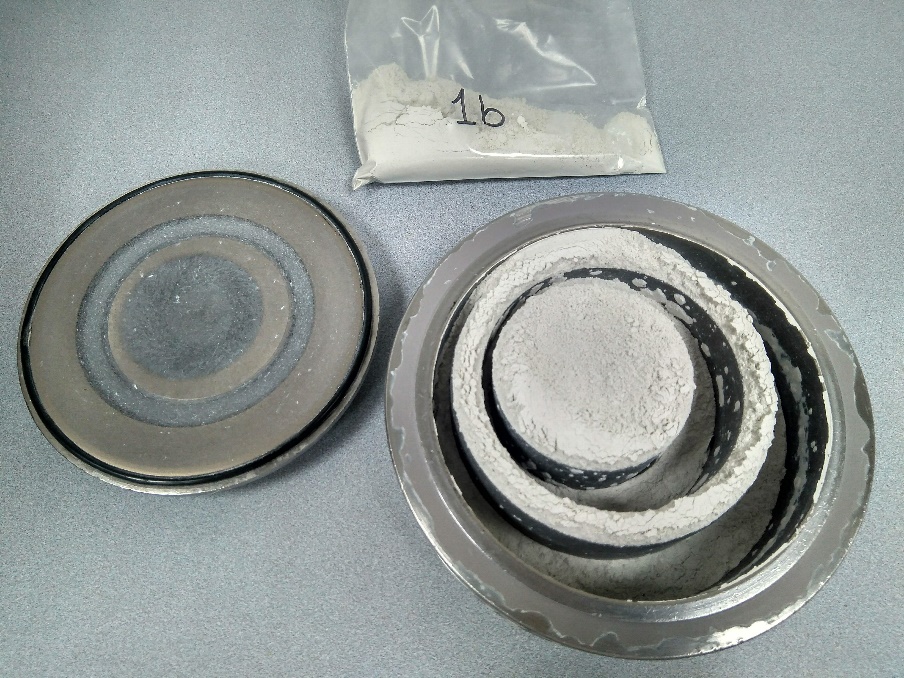

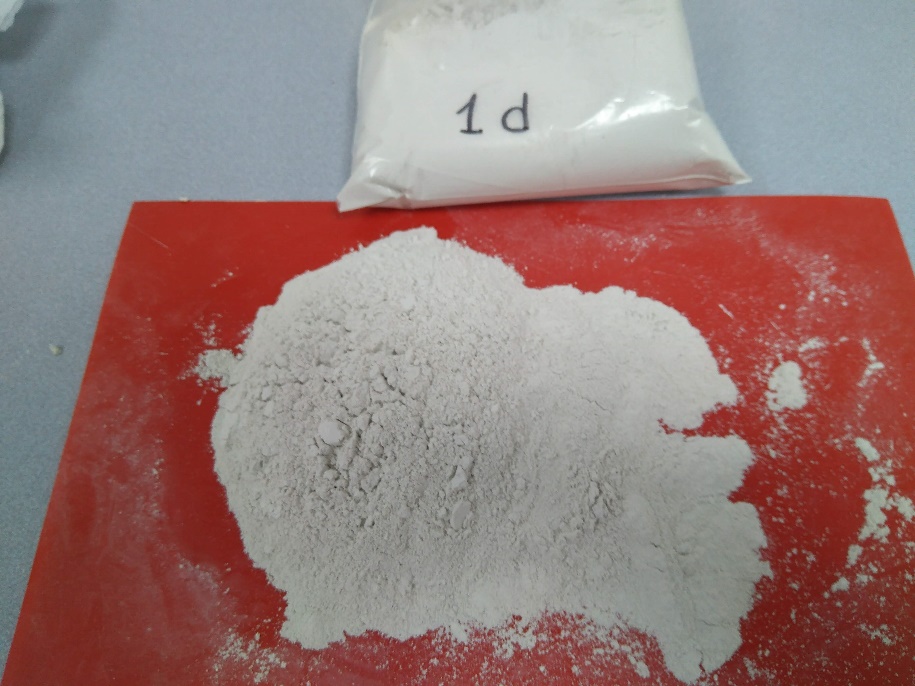


**(c)**

Figure S1: Images of (a) grinding to < 3 cm (b) grinding to < 1 cm and (c) milling machines.

Supplement: Supplementary file 1 [file molecules-25-01220-s001.zip › molecules-736556-SI/molecules-736556-SI.docx]
